# Predominant Role of mTOR Signaling in Skin Diseases with Therapeutic Potential

**DOI:** 10.3390/ijms23031693

**Published:** 2022-02-01

**Authors:** Fani Karagianni, Antreas Pavlidis, Lina S. Malakou, Christina Piperi, Evangelia Papadavid

**Affiliations:** 1 National Center of Rare Diseases—Cutaneous Lymphoma, Second Department of Dermatology and Venereal Diseases, Attikon University General Hospital, National and Kapodistrian University of Athens, 12462 Athens, Greece; karagiannifani@gmail.com (F.K.); antreas.pavlidis@gmail.com (A.P.); papadavev@yahoo.gr (E.P.); 2 Department of Biological Chemistry, Medical School, National and Kapodistrian University of Athens, 11527 Athens, Greece; linamalakou@gmail.com

**Keywords:** mTOR signaling pathway, psoriasis, atopic dermatitis, pemphigus, acne, cutaneous T cell lymphoma, melanoma, inhibitors, therapy

## Abstract

The serine/threonine kinase mechanistic target of rapamycin (mTOR) plays a pivotal role in the regulation of cell proliferation, survival, and motility in response to availability of energy and nutrients as well as mitogens. The mTOR signaling axis regulates important biological processes, including cellular growth, metabolism, and survival in many tissues. In the skin, dysregulation of PI3K/AKT/mTOR pathway may lead to severe pathological conditions characterized by uncontrolled proliferation and inflammation, including skin hyperproliferative as well as malignant diseases. Herein, we provide an update on the current knowledge regarding the pathogenic implication of the mTOR pathway in skin diseases with inflammatory features (such as psoriasis, atopic dermatitis, pemphigus, and acne) and malignant characteristics (such as cutaneous T cell lymphoma and melanoma) while we critically discuss current and future perspectives for therapeutic targeting of mTOR axis in clinical practice.

## 1. Introduction

Skin diseases affect millions of people worldwide and account for a notable proportion of the global burden of diseases. Over 4000 skin diseases and cutaneous adnexae constitute 15–30% of outpatient medical care and involve a large amount of diagnostic and therapeutic resources [1,2,3]. Of note, approximately 20% of patients requiring medical attention have skin symptoms while skin cancer screening accounts for 1.3% of suspicious skin findings, with 1% diagnosed as epithelial and 0.3% as melanocytic tumors [4]. Therefore, it is of primary importance to understand the physiological functions of skin cells, their interactions, as well as the underlying molecular mechanisms that lead to pathogenic function.

The skin lies at the interface of the environment with the internal milieu, offering a unique insight into health and disease. It is the largest organ of the human body, consisting of three different layers with distinct architecture and functions. It ensures defense against microbial attacks, chemical and physical insults, water or protein loss, and temperature control, all of which are essential elements for normal physiology and homeostasis [5]. Upon loss of skin protective functions, numerous genetic and acquired skin diseases occur, often associated with a wide spectrum of symptoms.

Recently, the advances in omics-based technologies have detected promising markers at genomic, transcriptomic, and metabolomic levels for skin disorders and they have shed light into molecular mechanisms and signaling pathways associated with their pathogenesis, aiming to discover key molecules that predict or prevent disease [6]. Research evidence has demonstrated that dysregulation of the mechanistic target of rapamycin (mTOR) signaling axis is involved in disease onset covering a broad spectrum of conditions from diabetes to various tumor types as well as inflammatory skin disorders and skin cancers.

Recent data indicate that mTOR is critically involved in skin cell growth, proliferation, and differentiation. Skin homeostasis and morphogenesis has been shown to rely on proper functioning of mTOR signaling to regulate the differentiation of keratinocytes and enable the epidermal stratification program along with hair follicles formation [7]. mTOR multiprotein complexes (mTORC1 and mTORC2) are required to produce a protective epidermal barrier, regulating in a distinct way the early stages and the terminal stages of epidermal differentiation [7,8].

Alterations in mTOR pathways can modulate protein synthesis, negatively affect cell growth and proliferation, and result in phenotypically diverse skin diseases. Keratinocytes were shown to deactivate Akt/mTORC1 signaling at the beginning of differentiation in healthy skin, using mTOR as a proliferation control [9]. Additionally, Akt was shown to be strongly activated in all epidermal layers of psoriatic lesions [8] and contribute to the rapid growth of psoriatic keratinocytes [10,11,12].

In the following sections, we discuss recent evidence on the implication of aberrant mTOR signaling in skin diseases, indicating its importance as a common denominator of skin disorders, and suggest potential therapeutic targeting options.

## 2. Structural and Biochemical Aspects of mTOR Signaling Axis

**Figure 1:**
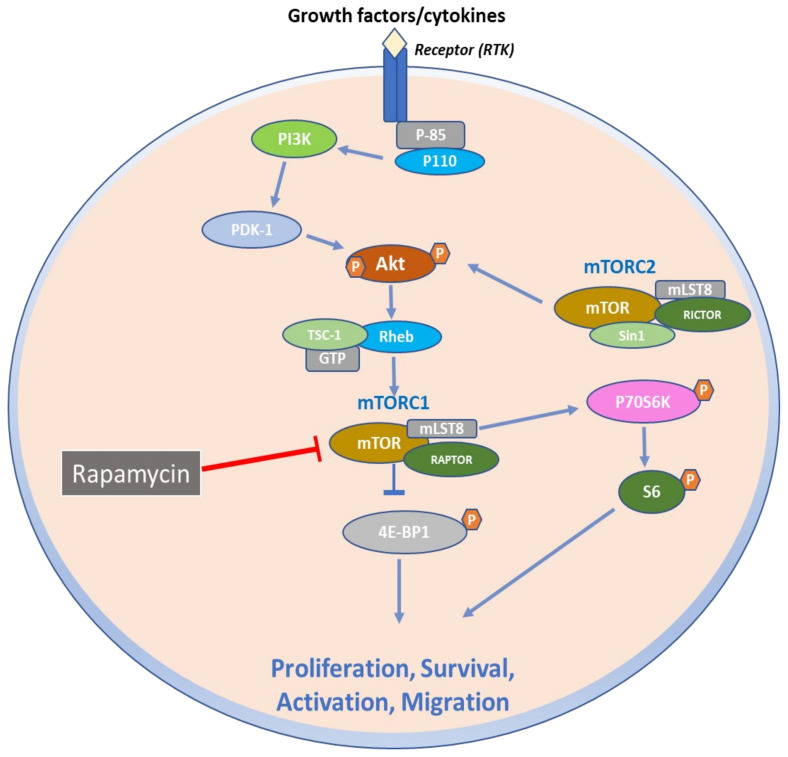
mTOR signaling axis in normal cell physiology. Upon receptor binding by growth factors and cytokines, phosphatidylinositol 3-kinase (PI3K) is activated and in turn phosphorylates AKT. AKT can phosphorylate and inactivate the tuberous sclerosis (TSC) tumor suppressor protein complex that acts as a GTPase-activating protein (GAP) for the RAS homolog enriched in brain (Rheb) small G protein to regulate its activity. Retention of the Rheb-GTP bound form activates mTOR, which is comprised of two main complexes that are associated with diverse proteins such as RAPTOR, mLST8, PRAS40 and DEPTOR for complex I (mTORC1), and RICTOR, mLST8, DEPTOR, mSin1, and PROCTOR for complex II (mTORC2). mTORC1 phosphorylates downstream p70S6 Kinase 1 and modulates the eukaryotic initiation factor 4E-binding protein (4E-BP1), which prevents it from hindering eIF4E, and enabling 40S ribosomal subunit to be recruited to mRNAs, leading to the initiation of protein translation. p70S6K also phosphorylates ribosomal protein S6 that is also involved in translational regulation by the 40S ribosomal subunit, thus regulating several cellular processes such as cell proliferation, activation, and survival. By contrast, mTORC2 regulated by growth factors phosphorylates distinct groups of proteins, enabling the regulation of actin cytoskeleton and cell migration.

The mechanistic target of rapamycin (mTOR) is a mammalian serine/threonine kinase of 289 kDa that functions as the catalytic subunit of mTOR complex 1 (mTORC1) and mTOR complex 2 (mTORC2) (Figure 1) [10]. Auxiliary protein ligand binding and varying rapamycin sensitivity, as well as diverse substrates and intracellular activities, distinguish these two complexes [11]. The mTORC1 is composed of three core subunits: mTOR, mammalian lethal with SEC13 protein 8 (mLST8), and the regulatory associated protein of TOR (RAPTOR) [12]. However, structural experiments have shown that mLST8 confers stability to the kinase domain of mTOR since removal of mLST8 affected only the activity of mTORC2 in mice and not of mTORC1 [13,14]. RAPTOR is required for appropriate mTORC1 subcellular localization and recruits mTORC1 substrates by binding to TOR signaling motifs found in mTORC1 substrates [15]. By binding to RAPTOR, the accessory factor PRAS40 (proline-rich AKT substrate 40 kDa) works as an endogenous mTORC1 inhibitor and regulates the activity of mTORC1 with DEPTOR (DEP-domain-containing mTOR-interacting protein) [16,17]. Further, mTORC2 is composed of three core subunits: mTOR, mLST8, and rapamycin-insensitive companion of mTOR (RICTOR). mLST8 contributes to the stability and function of mTORC2 complex, while RICTOR is essential for the binding of mSIN1 (MAPK-interacting protein 1) [13,18].

## 3. Function of mTOR Signaling Pathways

mTOR belongs to the PI3K/Akt signaling pathway and is implicated in hormone, growth factor and nutrient signaling, and is also critically involved in skin cell growth, proliferation, differentiation and motility [10,19,20,21,22].

Activation of mTOR pathway by environmental signals regulates anabolic processes and synthesis of necessary macromolecules for normal cell growth and proliferation. mTORC1 and mTORC2 set the biosynthetic machinery in motion to maintain abundance for the increased cellular requirements during cell mass accumulation through phosphorylation of substrates that are involved in the production of lipids, proteins, ATP, and nucleotides. Moreover, mTORC1 reduces autophagy of cellular components, leading to cell growth [23].

In regard to protein synthesis, mTORC1 phosphorylates and inhibits the eukaryotic initiation factor binding proteins (4E-BPs), leading to eIF4E release and promoting mRNA translation [20]. Additionally, mTORC1 phosphorylates S6K1, which enhances translation by activating eIF4B and degrading eIF4A inhibitor-programmed cell death 4 (PDCD4) [21,22]. Moreover, mTORC1 and S6K1 enhance the transcription of rRNA by increasing RNA polymerase I and III activity, regulating ribosome biogenesis, and protein synthesis, thus contributing to regulation of cell mass [23].

When cell size increases, mTORC1 boosts lipid biosynthesis. Activated mTORC1 induces the translocation of sterol regulatory element binding protein 1/2 (SREBP1/2) to the nucleus, where it upregulates, genes involved in de novo lipid and cholesterol production, key processes required for cell metabolism, nutrient signaling and cell growth [24]. In addition, mTORC1 is involved in lipid homeostasis genes expression controlled by peroxisome proliferator-activated receptor-γ (PPARγ), a key transcription factor in adipogenesis and metabolic homeostasis [25].

Moreover, mTOR signaling pathway regulates nucleotide abundance during cell growth. mTORC1 controls purine biosynthesis via the activation of ATF4 transcription factor as well as of mitochondrial tetrahydrofolate cycle enzyme methylenetetrahydrofolate dehydrogenase 2 (MTHFD2), which is a downstream target. mTORC1, also promotes pyrimidine biosynthesis mediated through carbamoyl-phosphate synthetase 2 activation and aspartate transcarbamoylase, dihydroorotase (CAD) [26,27].

Further, mTORC1 is also a critical regulator of autophagy. It facilitates the suppression of autophagy to avoid catabolism of newly synthesized cellular components and lysosome biosynthesis. There is evidence that different upstream signaling pathways regulate mTOR to inhibit or enhance autophagy levels depending on the cell requirements. Autophagy is mediated by the serine/threonine kinase, unc-51-like autophagy-activating kinase 1 (ULK1), which forms a complex with ATG13, ATG101, and FIP2000 [28]. When there is nutrient availability, mTOR-dependent phosphorylation of ATG13 inhibits ULK1 and prevents its activation by AMPK, which serves as a central activator of autophagy. In the presence of stress or reduced nutrients, mTORC1 is inhibited and ULK1 activity elevated, leading to autophagosome nucleation and induction of autophagy, allowing proteins and organelles to accumulate in growing cells [26,28].

mTORC2 is also involved in actin cytoskeleton reorganization, chemotaxis, and cell migration via the activation of PKCα [29], being further implicated in cell migration and metastasis of cancer cells [30].

Furthermore, mTORC2 associates with PDK1 to activate several PKC classes, the oncogene Akt and the ion transport regulator serum- and glucocorticoid-induced protein kinase 1 (SGK1) [11]. Akt is a key molecule in the PI3K pathway, which enhances proliferation and regulates insulin response, and appears to play a nodal role at the connection of metabolism to stress resistance through the transcription factors FOXO1/3a and NAD kinase [11]. Additionally, Akt controls the activity of glycogen synthase kinase 3b (GSK3b), suppressing apoptosis as well as modulating glucose homeostasis. Interestingly, Akt regulates mTORC1 and mTORC2 crosstalk through inactivation of the mTORC1 inhibitor and TSC2 as well as phosphorylation of the mSin1, which is a central factor of mTORC2 activity [31,32].

Consequently, alterations in PI3K/Akt/mTOR pathway can disrupt cellular homeostasis, alter protein synthesis, and skin cell growth, promoting inflammation and cancer growth.

## 4. Role of mTOR Signaling in Inflammatory Skin Diseases

### 4.1. mTOR Signaling in Psoriasis

Psoriasis is a widespread chronic inflammatory skin condition that affects approximately 2% of the global population. It is linked to a higher rate of morbidity and a lower quality of life [33]. Psoriasis vulgaris is the most prevalent clinical type of psoriasis, affecting 85–90% of all psoriasis sufferers [33]. Clinically, it is characterized by red inflammatory papules or plaques with copious and continuous white scales shedding in a localized or diffuse manner. The pathological features of the disease include parakeratosis, hyperkeratosis, papillary dermal capillary growth, acanthosis, and infiltration of the lesional skin with neutrophils, T lymphocytes, and other leucocyte types. It is therefore evident that the equilibrium between keratinocyte proliferation and differentiation is disrupted in psoriasis patients [34].

The signaling cascade of PI3K/Akt/mTORC1 has recently received interest as a critical homeostatic epidermal regulator with possible involvement in inflammatory disorders of the skin. Akt activation has been detected in the basal Ki-67+ proliferating cells as well as in all epidermal layers affected in psoriatic lesions [9,34]. This could be due to psoriatic keratinocytes maintaining their proliferation even after exiting the basal layer. As a result, epidermal keratinocyte hyperproliferation contributes to the development of psoriasis by keeping the PI3K/Akt/mTOR pathway upregulated. Akt, on the other hand, may suppress cellular apoptosis, which aids in the rapid growth of psoriatic keratinocytes [9].

**Figure 2:**
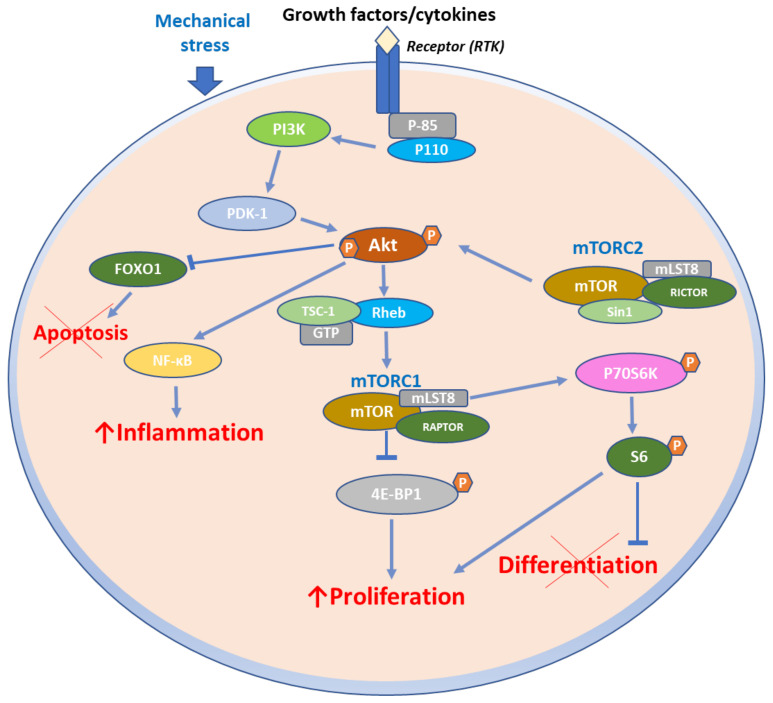
mTOR signaling in psoriasis. Several external stimuli (growth factors, cytokines, mechanical stress) are responsible for PI3K/AKT/mTOR overexpression and hyperactivation, which leads to activation of NF-κB inflammatory cascade and hyperproliferation, reduced epidermal differentiation, and apoptosis.

Furthermore, aberrant activation of the mTORC1 signaling axis was detected in all epidermal layers in the inflammatory milieu of psoriasis. A combination of cytokines (IL-1β, TNF-α, and IL-17A) was also shown to activate the mTORC1 signaling axis. The LncRNA maternally expressed gene 3 (MEG3) has been shown to enhance autophagy and suppress inflammation in TNF-α-treated keratinocytes and psoriatic mice via the PI3K/AKT/mTOR pathway [35]. Moreover, several deregulated miRNAs were demonstrated to activate the mTOR pathway in psoriatic skin [36,37], along with mechanosensitive molecules such as polycystins (Figure 2) [38].

#### mTOR Pathway in Psoriasis Immunopathogenesis

The pathophysiology of psoriasis is further complicated by the interplay of genetic, environmental, and immunologic variables, which is primarily marked by the interaction of innate and adaptive immune system [39].

The PI3K/Akt/mTOR pathway regulates both innate and adaptive immune responses [40] and is linked to the Th1/Th2/Th17 disequilibrium associated with psoriasis etiology [41]. The downstream targets (p70S6K and 4E-BP1) are activated by mTORC1 following protein synthesis and cell proliferation [42]. The “uncontrolled proliferation” of keratinocytes is the most crucial pathologic consequence in psoriasis [42]. mTOR signaling is activated by dysregulation of cytokines and growth factors, while the secretion of proinflammatory molecules (CXCL8, IL-6, and VEGF) by keratinocytes involves mTORC [43]. Furthermore, nucleophagy has been suggested as a key process that takes place during the differentiation and maturation of keratinocytes. Increased mTORC1 activity contributes to parakeratosis (nuclei retention), which is a psoriatic symptom [44,45].

Furthermore, mTOR axis is a critical regulator of the inflammatory and the proliferative aspects of psoriatic disease [40,41,46] mainly attributed to the significant role of mTORC1 and mTORC2 in immune cell energy metabolism and differentiation (Figure 2) [42]. mTORC1 signaling serves as a central modulator of the proliferation and differentiation of keratinocytes [47]. In support of this finding, Mitra et al. showed that IL-22 promotes growth of keratinocytes via the Akt/mTOR pathway [48]. PI3K activity was assessed in epidermal samples from normal skin, as well as in lesional and nonlesional psoriatic skin [49]. PI3K activity was increased by 6.7-fold in the epidermis of psoriatic plaques compared to normal skin. Moreover, Madonna et al. reported that phosphorylated Akt was elevated in lesional psoriatic skin in vivo as well as in activated psoriatic keratinocytes in vitro [8]. Both PI3K and Akt were further elevated in peripheral blood mononuclear cells (PBMCs) of patients with psoriasis compared to healthy subjects [50]. Ainali et al. conducted a large-scale study of gene expression in different samples of psoriatic skin and detected an overexpression of the PI3K/Akt pathway in plaque psoriatic skin [51].

Buerger et al. revealed that mTOR and the ribosomal S6 kinase, a downstream signaling protein, were activated in lesional psoriatic skin, indicating an association of mTOR signaling with psoriatic epidermal alterations [52].

Higher phosphorylated mTOR (p-mTOR) levels were detected in the basal layer. Further, increased S6K1 levels were detected in suprabasal layers in punch biopsies of patients with plaque psoriasis, suggesting the important role of mTORC1 in disease pathogenesis [34,52]. Shirsath et al. discovered that hyperactivated mTORC1 signaling plays a significant role in psoriasis. Photochemotherapy or Psoralen and ultraviolet light A (PUVA) treatment both improved the histology psoriasis score and normalized mTORC1 signaling in a hereditary mouse psoriasis model [53].

### 4.2. mTOR Signaling in Atopic Dermatitis

Atopic dermatitis is a chronic benign inflammatory manifestation of the epidermis that involves skin lesions and susceptibility to cutaneous infections. Epidermal barrier dysfunctions are attributed to dysregulation of keratinocytes’ terminal differentiation, which allows the entrance of cutaneous antigens and triggering of eosinophils and Th2 cells. This is followed by chemokine and cytokine activation, leading to their infiltration and accumulation in the epidermis to initiate atopic dermatitis. IL-4, IL-9 and IL-13 cytokines represent some of the main effectors in the pathogenesis of atopic dermatitis [54].

However, cytokine-induced proliferation mechanisms of keratinocytes in atopic dermatitis remain elusive. mTOR activation participates in the overgrowth of keratinocytes as a mediator of their communication with the immune system. IL-13 activates the mTOR/mir-143 axis, which is followed by downregulation of IL-13a1, leading to an enhancement loop of IL-13 function and downregulation of epidermal barrier-related proteins. Preclinical studies of IL-13-stimulated HaCaT cells have shown that rapamycin can mediate this pathway and ease the effects of IL-13 in atopic dermatitis [55].

Additionally, mTOR is a modulator of autophagy through the AMPK pathway. IL-37 can reverse the manifestation of inflammation in atopic dermatitis by regulating the microbiota and the autophagy signaling pathway via mTOR. IL-37 downregulates the expression levels of mTOR by increasing the AMPK levels, leading to enhanced autophagy and reduction of the expression of inflammatory cytokines such as IL-31 and IL-33 [56].

Furthermore, some patients bearing mutations in filaggrin, a filament aggregating protein, exhibit a high predisposition for atopic dermatitis. Filaggrin is a protein crucial for the normal function of the skin barrier and, upon dysregulation, leads to vulnerability of the epidermal structure [57]. Previous studies have shown that mTORC1 overexpression correlates with a reduction in filaggrin expression [58].

### 4.3. mTOR Signaling in Pemphigus

Pemphigus vulgaris (PV) is an autoimmune chronic blistering skin disease that involve mucous membranes as well as the skin. Intraepidermal blisters characterize the disease along with autoantibodies against desmosomal cadherin desmoglein (Dsg) 1 and 3 [59,60].

Nondesmoglein autoantibodies including acetylcholine receptors, antimitochondrial autoantibodies, and E-cadherin can also be present in the serum of PV patients [60]

Acantholytic PV cells were shown to activate apoptosis in a relevant study [61]. P-mTOR has been detected at the basal cells of pemphigus vulgaris IgG (PV IgG)-injected mice but not in a scattered localization observed in control mice injected with normal human serum. Mice injected with PV IgG and further treated with rapamycin did not exbibit suprabasal acantholysis, indicating the implication of mTOR in the development of PV lesions [62]. The activation of the mTOR pathway (PI3K/AKT/mTOR/p70S6K) has recently been linked to dysregulation of T helper 2/regulatory T (Th2/Treg) cell balance in PV patients’ peripheral blood. Rapamycin was demonstrated to block the differentiation of Th2 cell while promoting Treg cells in vitro, indicating a connection between the activity of mTOR pathway and the imbalance of Th2/Treg cells in PV [63].

This study points towards a new therapeutic strategy for PV that involves blocking or interference with the signaling pathway and associated enzymatic mechanisms that induce blistering.

### 4.4. mTOR Signaling in Acne

Acne is a persistent cutaneous inflammatory condition with a multifactorial origin. Hyperseborrhea, abnormal keratinization of the pilosebaceous duct, *Cutibacterium acnes* (*C. acnes*) colonization, and inflammation are all important contributors to acne pathophysiology [64].

Recently, it was demonstrated that insulin signaling stimulates the PI3K/Akt pathways along with mTOR in sebocytes, resulting in increased synthesis of proteins and lipids, cell proliferation, and inflammation [65]. It was further shown that 5-aminolevulinic acid photodynamic therapy (ALA-PDT) inhibits sebocyte growth via the mTOR-p70 S6K signaling pathway and lowered lipogenesis via the mTOR-SREBP-1/PPAR signaling axis. The reduction of lipogenesis and sebocyte growth after ALA-PDT may be responsible for sebaceous gland atrophy and reduced sebum output [66]. Another group has shown that mTOR gene expression is 17.96- and 20.77-fold higher in nonlesional (NLS) and lesional skin (LS), respectively, from 10 acne patients when compared to skin samples from healthy subjects. It was observed that during the transition from healthy state to lesional skin, mTOR expression steadily increased [67].

**Figure 3:**
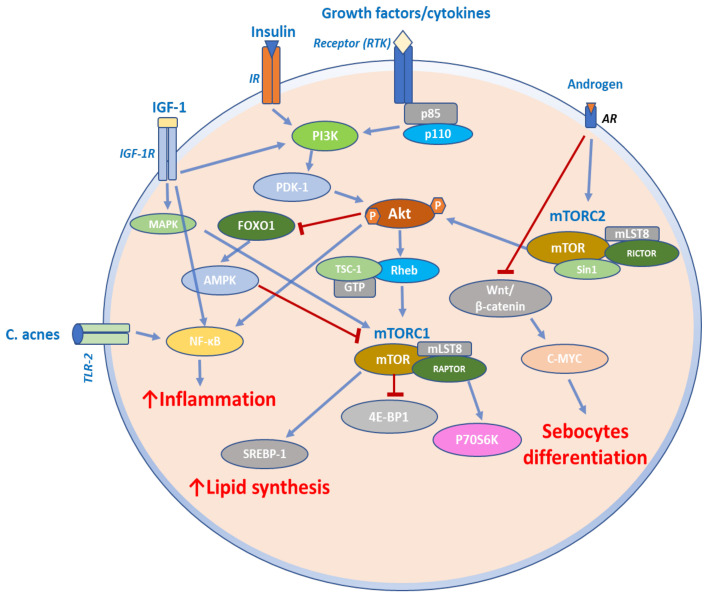
mTOR signaling in acne. Growth factors, insulin, and IGF-1 induce lipid synthesis by increasing SREBP-1 expression via the PI3K/Akt/FoxO1/mTORC1 pathway and the MAPK pathway. IGF-1 can also induce proinflammatory cytokine expression in sebocytes through NF-κB activation. Virulent C. acnes binds to TLR-2 and activates the NF-κB pathway to induce an inflammatory response in sebocytes. Androgens stimulate altered sebocytes differentiation through mTORC2 and by Wnt/β-catenin signaling pathway.

Furthermore, mTOR, FoxO1, and serum IGF-1 along with a high-glycemic-load diet may be involved in the development of acne (Figure 3) [67]. A study of 40 acne patients and 20 controls showed that the patients exhibited considerably higher serum IGF-1 levels than controls. The cytoplasmic FoxO1 expression was much higher in acne patients than in controls, which exhibited nuclear expression in lesional skin biopsies. In patients with acne, both cytoplasmic and nuclear mTOR expression was considerably higher than in controls. Furthermore, greater IGF-1 blood levels and cytoplasmic expression of FoxO1 and mTOR were significantly linked with a high glycemic load diet as assessed by a diet questionnaire.

Melnik et al. revealed that insulin-IGF-1 signaling induced the extrusion of FoxO1 to the cytoplasm, but nuclear FoxO1 decreased hepatic IGF-1 production, impairing somatic growth [68]. FoxO1 inhibited androgen signaling and interacted with proteins involved in the regulation of sebum lipogenesis. It is involved in modulation of innate and adaptive immunity, protecting against oxidative stress, while acting as an mTORC1 rheostat [68]. In this way, FoxO1 connects nutrients availability to mTORC1-driven processes such as cell differentiation, hyperproliferation of acroinfundibular keratinocytes, hyperplasia of sebaceous glands, enhanced sebaceous lipogenesis, elevated BMI, and insulin resistance. By upregulating the FoxO1 transcription factor, the mTORC signal was engaged in the therapeutic response to isotretinoin or metformin treatment for acne [68,69,70]. Isotretinoin also increased p53 expression, which further upregulated FoxO1 and PTEN expression while suppressing the IGF-1 levels and androgen receptors, leading to decreased IGF-1/mTORC1 and androgen signaling (Figure 3) [68,71,72].

All the above findings support the idea that mTOR axis is implicated in the pathophysiology of acne. Since a high glycemic load diet is linked to mTOR overexpression, further understanding of the impact of the Western diet on acne and evaluation of the therapeutic response to acne treatment is required.

## 5. Role of mTOR Signaling in Skin Cancer

### 5.1. mTOR Signaling in CTCL

Cutaneous T-cell lymphomas (CTCL) are heterogeneous and rare non-Hodgkin lymphomas, characterized by malignant T lymphocytes infiltrating the skin. Mycosis fungoides (MF) is the common form of CTCL (~55%), which arises from effector memory T-cell residents in skin and is characterized by slow progression from patches to plaques and eventually to tumors. MF presents a five-year overall survival (OS) of 74.5% and median survival of 18.3 years. Sézary syndrome is less frequent (~5%) but more aggressive than MF. It originates from central memory T-cell residents in skin and is characterized by atypical malignant Sézary cells in blood, lymph nodes, and skin. Sézary syndrome presents a five-year OS of 26% with a median survival of ~3 years [73].

The anabolic demands of proliferating cancer cells require adaptation of metabolic profile, increasing glycolytic rate (Warburg effect), which may be attributed to mTOR pathway. The investigation of mTOR activity in CTCL cell lines revealed the constitutive activation of mTOR in MyLa, HUT78, and SeAx cells. Specifically, phosphorylation of p70S60 in CTCL cell lines indicates the constitutive activation of mTORC1, and associated signaling pathway, in a cytokine-independent manner [74]. Additionally, the same cell lines present growth inhibition upon treatment with rapamycin (5 nM). However, rapamycin has not been shown to induce apoptosis, instead it causes cell cycle arrest in G1/G0 in CTCL cell lines [74].

The proangiogenic vascular endothelial growth factor (VEGF) implicated in most hematological malignancies has also been detected in CTCL biopsies and respective cell lines [75]. The most important VEGF regulator is the hypoxia-inducible factor 1a (HIF-1a), which can be regulated by the mTOR pathway. The use of rapamycin in xenograft animal models has been shown to significantly reduce tumor growth, indicating that mTOR is a promising drug target for CTCL therapy [74]. Further studies in murine and human T-cell lymphomas demonstrated that rapamycin suppressed tumor growth, decreased mTOR activity, and reduced S6K and S6 phosphorylation [76]. The most surprising evidence is that Akt showed reduced phosphorylation in serine 473, which is the downstream target of mTORC2 [76].

Additionally, the inhibition of mTOR via rapamycin treatment altered the metabolism of lymphoma cells, decreasing the glycolytic state and provoking regression of Warburg effect in cancer cells. Rapamycin-treated cells and xenograft models exhibited decreased expression of glucose transporter (GLUT3), lactate dehydrogenase a (LDHA), and hexokinase 2 (HK2) and thus provide evidence of the importance of mTOR signaling in aerobic glycolysis [76]. The mTOR pathway is suggested to link the metabolic state with T cell differentiation and activation. The activation of T cells was required for the upregulation of glycolytic activity of lymphocytes via mTOR signaling, whereas downregulation of this process induced the regulatory T cell phenotype [77].

Although lymphoma cells treated with rapamycin display reduced utilization of glucose, they enhance resistance in low-glucose conditions that stimulate the tumor microenvironment [26,27,28]. However, rapamycin exerts tumor suppressive effects in CTCL xenograft model and lymphoma cells display increased resistance against treatment, which is attributed to the switch of their function towards mitochondrial metabolism and oxidative phosphorylation. This evidence indicates that oxidative phosphorylation is a potential target for CTCL, leading to an adjuvant therapeutic outcome that requires further investigation [76].

Another study addressing the importance of TOX (thymocyte selection-associated high mobility group box) in CTCL demonstrated that upon TOX knockdown the cell viability of HuT78 cell lines was inhibited. KEGG pathway analysis revealed the implication of mTOR pathway in CTCL, as well as the therapeutic potential of mTOR inhibitors [78].

### 5.2. mTOR Signaling in Melanoma

Melanoma is a skin cancer type caused by dysregulation of melanocytes, located in the epidermis junction, synthesizing and producing melanin, a photoprotection protein. Approximately, 50% of melanoma cells have a mutated copy of *BRAF* oncogene [79]. *BRAF* gene encodes the BRAF serine/threonine kinase, which upregulates the signaling pathway of mitogen-activated protein kinase (MAPK). Melanoma patients bearing the serine/threonine kinase BRAF mutation treated with BRAF and MEK inhibitors exhibited a 70% response rate. Moreover, the BRAF mutation is present in almost 50% of melanoma cases, and in more than 90% cases, BRAF harbors the V600E point mutation [80]. Targeting the MAPK pathway using a combination of BRAF and MEK inhibitors faces one significant barrier. Melanoma cells acquire resistance in therapies by rewiring the signaling pathways. This can be achieved by activating mutations in NRAS or alternative activation of RTK (RAS/RAF/MEK/ERK)-mediated pathways [81]. The BRAFV600E mutation and PI3K/PTEN/mTORC1 have a synergistic action in refractory and metastatic melanoma, which is AKT-independent. A recent study showed that BRAF/MAPK and PI3K/mTORC1 regulate cooperatively the activation of 4E-PB1 p70S6K, ribosomal protein S6, and mTORC1 downstream targets [82].

Primary benign and malignant melanomas progress to invasive stage rapidly, while this transition from primary to more aggressive invasive melanomas and metastatic forms has been associated with Akt/mTOR activation. Studies have shown that in invasive metastatic melanomas, Akt serves as a molecular switch leading to upregulation of mTOR and of the downstream target, S6K1 [82].

Furthermore, recent studies reveal the implication of signaling PI3K/mTOR axis in the pathogenesis of melanoma. In early-stage melanomas, tumor cells start to grow vertically and transit to an invasive phase, leading to distant metastasis. This transition from primary melanoma to metastatic type has been associated with Akt activation, acting as a link between signals and mTOR/S6K1 upregulation, while enhancing angiogenesis, which further nurtures the aggressiveness of metastatic melanomas.

Table 1 provides a summary of key findings of main studies supporting the implication of mTOR signaling in skin diseases.

## 6. Therapeutic Targeting of mTOR Signaling Axis in Skin Diseases

Taking all this information into account, it is evident that mTOR signaling is implicated in most skin disorders, mainly affecting cell proliferation. To this end, targeting mTOR signaling presents a promising therapeutic approach with a variety of mTOR inhibitors being currently tested in skin disorders.

### 6.1. Role of mTOR Inhibitors in Psoriasis

The use of mTOR inhibitors in psoriasis has been encouraged by studies indicating the upregulation of PI3K/Akt/mTOR pathway in psoriatic plaques and around the lesional skin [83,84,85,86]. Despite the fact that inhibitors of mTOR have been employed in the treatment of various dermatologic disorders such as Kaposi sarcoma, tuberous sclerosis, Muir-Torre syndrome, and neurofibromatosis [83], their effects on psoriasis have been investigated only in a small number of case reports [84,86].

Rapamycin, a first-generation mTOR inhibitor, has been studied in psoriasis patients for its antiproliferative and immunosuppressive characteristics. Everolimus (a sirolimus derivative) was demonstrated to be successful in a single patient [85], whereas sirolimus in combination with cyclosporine therapy was found to be effective in a bigger study [86]. Furthermore, everolimus was shown to improve skin lesions in a kidney transplant patient with resistant psoriasis [87]. Furthermore, since the vitamin D analog 1a, 25-dihydroxyvitamin D3-3-bromoacetate (BE) has been demonstrated in vitro to reverse IL-22-induced psoriasiform alterations [39], suppression of the PI3K/Akt axis has been suggested as a possible treatment method. Topical rapamycin therapy resulted in a considerable improvement in the clinical score, although plaque thickness remained unaltered in a limited clinical trial [84]. To learn more about this treatment approach, a study investigated the efficacy of topical administration of rapamycin in a murine imiquimod-induced psoriasis model, which exhibited mTORC1 signaling activation similar to human psoriasis [88,89]. In comparison to the control group, mice treated with rapamycin demonstrated a considerable amelioration of their clinical appearance (redness, flaking, and swelling) as well as reduced angiogenesis and epidermal thickness normalization. mTORC1 and downstream molecules were clearly activated in imiquimod-treated mice, whereas rapamycin lowered their activity in untreated mice. Cell differentiation markers including involucrin, keratins, and loricrin exhibited normalized expression and distribution after rapamycin. In addition, rapamycin therapy inhibited innate immune cells from entry into the draining lymph nodes [89]. Rapamycin therapy also restored tropomyosin expression in the same mouse model, which is downregulated in psoriatic lesions and contributes to illness [90]. In conclusion, supporting evidence indicates that mTORC inhibitors applied topically may prove to be an efficient antipsoriatic therapeutic method and requires further investigation.

### 6.2. Role of mTOR Inhibitors in CTCL

An increasing number of drugs targeting mTOR pathway has been investigated for the treatment of several malignancies, including CTCL. The first-generation mTOR inhibitors, rapamycin, and its analogs showed limited antitumor effects due to the feedback mechanism, which involves mTORC2. The second-generation mTOR inhibitors were demonstrated to overcome this feedback mechanism by simultaneously blocking both mTORC1 and mTORC2, while recently, a third generation of compounds known as Rapa-Links have been investigated that contain a rapamycin-FRB-binding element coupled to the mTOR inhibitor [91,92,93].

The low efficacy of first- and second-generation mTOR inhibitors in preclinical studies may be attributed to the upregulation of several crosstalk pathways, such as RAS/RAF/MAPK/ERK and NF-κB, that work in parallel with mTOR inhibition [31,32]. Dual PI3K/mTORC1/2 inhibitors may lead to better antitumor effects, as they can simultaneously block the mTOR pathway and affect the parallel pathways. A dual PI3K/mTORC1/2 inhibitor has been shown to maintain antitumor effects in patient-derived Sézary cells ex vivo as well as in a xenograft mouse model. Specifically, PF-502, a dual PI3K/mTORC1/2 inhibitor administered in CTCL cell lines, was shown to induce arrest of cell cycle and apoptosis [94]. Additionally, PF-502 has demonstrated excess antitumor and apoptotic effects in patient-derived Sézary cells ex vivo, while it is less apoptotic in nonmalignant cells [94]. Moreover, PF-502 exerted antitumor and apoptotic activity in a mouse xenograft model of CTCL. Interestingly, PF-502 presents a greater apoptotic effect in vivo rather than in vitro, suggesting that PF-502 indirectly inhibits tumor growth, possibly by affecting the tumor microenvironment [94].

### 6.3. Role of mTOR Inhibitors in Melanoma

Recent studies provide evidence that mTOR inhibitors may represent a supplementary weapon in the quiver of melanoma treatment alongside with a combination of BRAF and MEK inhibitors [80]. Targeting AKT/mTOR and MAPK pathway at the same time, was shown to reverse the resistance of melanoma cells and further propose a new approach for melanoma treatment.

Preclinical studies using mTORC1 inhibitors such as rapamycin and RAP analogs, everolimus, and temsirolimus, have shown inhibition of tumor growth and invasion in several melanoma cell lines [95]. However, these agents have had multiple adverse side events in clinical studies [96].

As previously stated, RAS and MEK mutations contribute to treatment resistance in aggressive melanoma. In vitro experiments have shown that combining BRAF inhibitors with PI3K/mTOR inhibitors overcomes this resistance [97]. Another suggested pathway for rewiring the resistance and the aggressiveness of melanoma is suppressing both mTOR and autophagy. A specific mTOR inhibitor, temsirolimus, exhibits synergistic action with hydroxychloroquine, an autophagy inhibitor, leading to cell death via apoptosis in melanoma cells [98].

Another effective strategy for treating melanoma may be the combination of 17AAG, an HSP90 inhibitor with NVP-BEZ235, a PI3K/mTOR inhibitor. HSP90 is a chaperone, mediating protein 3D structure and function. Monotherapy with HSP90 inhibitors faces poor therapeutic results in carcinogenesis. However, when combined with other agents, such as mTOR inhibitors, it enhances antitumor activity [99].

All key studies addressing the therapeutic approaches targeting mTOR signaling in skin diseases are summarized in Table 2.

## 7. Conclusions—Future Perspectives

Taken together, there is significant evidence that the mTOR signaling axis is highly implicated in the pathogenesis of skin disorders including inflammatory and proliferative disorders as well as tumor formation. The implication of mTOR pathways in pathogenic skin conditions may be either direct or indirect through crosstalk with other signaling pathways including MAPK, PTEN, AMPK, and PKC. A deep understanding of the regulation of this complex network is essential to improve current targeting options and strategies. Small synthetic compounds have been investigated both alone or in combination with other agents in vitro and in vivo models, demonstrating either a potential for topical application such as onto psoriatic skin or for oral administration, with significant antitumor effects in CTCL and melanoma. Some agents have been administered in clinical trials with effective responses and clinical outcomes. However, several mTOR inhibitors exhibit adverse side effects and limitations, indicating that further research into their selection and the development of novel safe but potent agents is required.

## Figures and Tables

**Table 1:** Studies indicating the implication of mTOR signaling in skin diseases.

Disease	Study	Type of Study/Sample Type/Main Findings	Reference
**Psoriasis**	Pike et al., 1989	**Skin biopsies**: PI3K activity was increased by 6.7-fold in the epidermis of psoriatic plaques compared to normal skin.	[49]
	Calautti et al., 2005	**In vitro**: Kinase activities of PI3K and Akt are induced, Akt involved in suppression of cell apoptosis.	[9]
	Ochaion et al., 2009	**PBMCs**: PI3K and Akt were elevated in PBMCs of patients with psoriasis compared to healthy subjects.	[50]
	Ainali et al., 2012	A **large-scale study** of gene expression in different samples of psoriatic skin detected an overexpression of the PI3K/Akt pathway in plaque psoriatic skin.	[51]
	Mitra et al., 2012	**In vitro**: IL-22 promotes growth of keratinocytes via the Akt/mTOR pathway.	[48]
	Buerger et al., 2017	**In vitro and punch biopsies**: Akt activation detected in basal Ki-67+ proliferating cells as well as in all epidermal layers affected in psoriatic lesions.	[34]
	Madonna et al., 2012	**In vitro and skin biopsies**: Akt was strongly active in all epidermal layers of psoriatic lesions; phosphorylated Akt was elevated in lesional psoriatic skin in vivo as well as in activated psoriatic keratinocytes in vitro.	[8]
	Buerger et al., 2013	**Clinical**: higher p-mTOR levels were detected in the basal layer along with increased S6K1 in suprabasal layers in punch biopsies of patients with plaque psoriasis, suggesting the important role of mTORC1 in disease pathogenesis.	[52]
	Xu et al., 2017	**In vitro**: miR-155 knockdown led to a significant decrease in cell proliferation; the expression of several apoptosis-related factors was dramatically changed, such as PTEN, PIP3, AKT, p-AKT, Bax and Bcl-2. **Clinical**: miR-155 mRNA expression was up-regulated in psoriasis tissues compared with adjacent noncancerous tissues.	[36]
	Rongna et al., 2018	**In vitro**: miR-876-5p restrains proliferation, cell cycle, cell invasion and adhesion in psoriatic cells. **Clinical**: low-level of miR-876-5p in psoriatic tissues and blood compared to the respective normal samples.	[37]
	Gargalionis et al., 2018	**In vitro**: PC1 knockdown in HaCaT cells led to an elevated mRNA expression of psoriasis-related biomarkers Ki-67, IL-6, TNF-α, VEGF and Bcl-2; PC1 functional inhibition was accompanied by increased cell proliferation and migration of HaCaT cells.	[38]
**Atopic Dermatitis**	Jia et al., 2020	**In vitro**: IL-13 increased the expression levels of p-mTOR, p-S6K1, and p-Akt.	[55]
**Pemphigus vulgaris**	Grando et al., 2009	**In vivo**: p-mTOR detected at the basal cells of PV IgG injected mice compared to a scattered localization observed in control mice injected with normal human serum.	[62]
	Lai et al., 2021	**Clinical**: PV patients showed elevated serum IL-4 when compared with HCs, and serum IL-4 level was positively correlated with the titer of anti-Dsg1/3 antibody and disease severity; elevated mRNA levels of PI3K, AKT, mTOR and protein levels of PI3K (P85), AKT, p-AKT (Ser473), mTOR, p-mTOR (Ser2448), p-p70S6K (Thr389), GATA3; reduced protein of forkhead box protein 3.	[63]
**CTCL**	Kremer et al., 2010	**In vitro**: Constitutive activation of mTOR kinase in MyLa, HUT78, SeAx and MK-1; rapamycin induced cell cycle arrest in G1 phase and delayed cell growth of CTCL cell lines and primary CD4+ cells isolated from Sézary patients; rapamycin treatment inhibits mTOR, which regulates HIF-1α and consequently decreases VEGF expression in CTCL cell lines. **In vivo**: Rapamycin treatment delays tumor growth in MyLa xenotransplant model.	[74]
	Krejsgaard et al., 2006	**In vitro**: VEGF expression in MyLA and SeAx cell lines regulated by mTOR signaling. **In vivo**: VEGF expression in dermal lesions of different stages of CTCL patients through mTOR regulation.	[75]
	Kittipongdaja et al., 2015	**In vitro**: Rapamycin suppressed tumor growth and mTOR activity in MBL2, HH and Hu78 cell lines. Additionally, rapamycin-treated MBL2, HH, and Hu78 cell lines exhibited reduce aerobic glycolysis and decreased glucose utilization. **In vivo**: Rapamycin treatment demonstrated suppression of tumor growth and reduce tumor mass in CTCL xenotransplant model.	[76]
	Shi et al., 2011	**In vitro**: mTOR, via HIF-1α dependent transcriptional program, mediated glycolytic activity and contributed to the lineage selection between Th17 and Tregs.	[77]
	Xu et al., 2020	**In vitro**: Pathway analysis revealed mTORC1 activation in CTCL cell lines; rapamycin inhibited mTORC1 signaling and restrain the growth of CTCL cells.	[78]
**Melanoma**	Wang et al., 2021	**In vitro**: BRAF/MEK inhibitors combination restored mTORC1 activity, in resistance-associated mTORC1 signaling melanoma cells.	[80]
	Shao et al., 2015	**In vitro**: BRAF/MAPK and PI3K/mTORC1 regulated cooperatively the activation of 4E-PB1 p70S6K, ribosomal protein S6 and, mTORC1 downstream targets..	[82]
		**In vivo**: The transition of primary benign and malignant melanomas progression to invasive stage was associated with Akt/mTOR activation.	[82]

**Table 2:** Therapeutic approaches that target mTOR signaling in skin diseases.

Disease	Drug Name/Approach	Type of Study/Effects	Reference
**Psoriasis**	Everolimus combined with cyclosporin	**Case report** (psoriasis patient) − reduced desquamation − decreased the thickness of lesions − 60% decrease in PASI score and immunosuppressive characteristics	[85]
	Sirolimus combined with cyclosporin	**Phase 2 randomized controlled trial** (N = 150) − reduction in their respective toxicities, notably cyclosporine-induced nephrotoxicities − antiproliferative and immunosuppressive characteristics	[86]
	Everolimus combined with tacrolimus	**Case Report** (renal transplant patient with psoriasis) − synergistic effect of everolimus and tacrolimus − resolution of the recalcitrant psoriatic manifestations	[87]
	Sirolimus	**In vitro** − sirolimus can penetrate human skin **(Franz cell assay in stratum corneum)** **Clinical study** (N = 24) − clinical score improvement − plaque thickness remained unaltered	[84]
	Rapamycin	**In vivo** (murine imiquimod-induced psoriasis model) − ameliorated clinical appearance (redness, flaking and swelling) − reduced angiogenesis and epidermal thickness normalization − normalized expression and distribution of cell differentiation markers (involucrin, keratins, and loricrin) − inhibited innate immune cells from entry into the draining lymph nodes	[89]
	Rapamycin	**In vitro** − reversed the expression of TPM1 and TPM2 in a cell model of psoriasis induced by TNF-α − inhibited cell proliferation of HaCaT and HEK cell model of psoriasis − restores the cell cytoskeleton and reduction of the number of cells in S phase of HaCaT or HEK cell model of psoriasis **In vivo** − ameliorated skin condition in an animal model of psoriasis induced by IMQ − reversed the expression and methylation of TPM1 and TPM2 in an animal model of psoriasis **Preclinical study** (Normal = 20 cases, lesional = 20 cases and nonlesional tissue samples = 20 cases) from normal individual or patients with psoriasis) − TPM1 and TPM2 expression were downregulated in psoriasis tissues with higher methylation level	[90]
**Atopic Dermatitis**	Rapamycin	**In vitro** − can ease the effects of IL-13 in atopic dermatitis	[55]
**Pemphigus vulgaris**	Rapamycin	**In vivo** − mice injected with PV IgG and further treated with rapamycin did not exhibit suprabasal acantholysis	[62]
	Rapamycin	**In vitro** − blocked the differentiation of Th2 cell, promoting Treg cells	[63]
**CTCL**	PF-502	**In vitro** − induced cell cycle arrest − increased apoptotic activity	[94]
	PF-502	**Xenograft mouse model** − inhibited tumor growth, possibly by affecting the tumor microenvironment − increased apoptotic activity	[94]
**Melanoma**	Rapamycin combined with NVP-BEZ235	**In vitro** − induced cell cycle arrest and apoptosis in melanoma cell lines	[95]
	Everolimus	**In vitro** − inhibited tumor growth and invasion in several melanoma cell lines	[95]
	Temsirolimus	**In vitro** − induced autophagy	[98]
	Rapamycin combined with BAY43-9006	**In vitro** − inhibited melanoma cell proliferation	[95]
	GSK2118436 combined with GSK1120212	**In vitro** − overcame B-RAF inhibitor resistance − inhibited cell growth, decreases ERK phosphorylation, decreased cyclin D1 protein, and increased p27(kip1) protein	[97]
	GSK2118436 combined with GSK1120212 and GSK2126458	**In vitro** − enhanced cell growth inhibition and decreased S6 ribosomal protein phosphorylation	[97]
	Combination of the lysosomotropic agent and autophagy inhibitor hydroxychloroquine (HCQ) with temsirolimus	**In vitro** − led to melanoma cell death via apoptosis **3D spheroid cultured and tumor xenografts** − suppressed melanoma growth and induced cell death	[98]
	HSP90 inhibitor 17AAG with the PI3K/mTOR inhibitor NVP-BEZ235	**In vitro** − decreased melanoma cell growth, inducing apoptosis and targeting simultaneously the MAPK and PI3K/AKT/mTOR pathways	[99]

## Abbreviations

mTOR	Mechanistic Target of Rapamycin
PI3K	Phosphatidylinositol 3-kinase
Akt	Ak strain transforming
mTORC1	mechanistic target of rapamycin complex 1
mTORC2	mechanistic target of rapamycin complex 2
mLST8	mammalian lethal with SEC13 protein 8
RAPTOR	Regulatory-associated protein of mTOR
PRAS40	proline-rich Akt substrate of 40KDa
DEPTOR	DEP-domain containing mTOR-interacting protein
RICTOR	rapamycin-insensitive companion of mTOR
mSIN1	mammalian stress-activated protein kinase-interacting protein
MAPK	Mitogen-activated protein kinase
4E-BPs	eukaryotic translation initiation factor 4E (eIF4E)-binding protein 1
PDCD4	Suppression of Programmed Cell Death 4
elF4E	Eukaryotic translation initiation factor 4E
S6K1	S6 kinase 1
rRNA	ribosomal RNA
SREBP1/2	Sterol regulatory element-binding proteins
PPARγ	Peroxisome proliferator-activated receptor γ
ATF4	Activating transcription factor 4
MTHFD2	Methylenetetrahydrofolate dehydrogenase (NADP+ dependent) 2
CAD	Dihydroorotase
ULK1	Unc-51 Like Autophagy Activating Kinase 1
ATG13	Autophagy-related gene 13
PKCα	Protein kinase Cα
PDK1	Phosphoinositide-dependent protein kinase
PKC	Protein kinase C
SGK1	Serum and glucocorticoid-regulated kinase 1
FOXO1/3a	Forkhead transcription factor forkhead box protein O1/3a
NAD	Nicotinamide adenine dinucleotide
GSK3b	Glycogen synthase kinase 3 beta
TSC2	Tuberous Sclerosis Complex 2
IFN	Interferon
mDCs	Myeloid dendritic cells
Th-1	Type 1 T helper cells
Th-22	Type 22 T helper cells
Th-17	Type 17 T helper cells
TNF-α	Tumour Necrosis Factor alpha
IL-17	Interleukin 17
miRNAs	microRNA
p70S6K	70-kDa ribosomal protein S6 kinase
CXCL8	C-X-C motif ligand 8
VEGF	Vascular endothelial growth factor
PBMCs	Peripheral blood mononuclear cells
p-mTOR	Phosphorylated mTOR
PUVA	Psoralen and ultraviolet light A
AD	Atopic dermatitis
AMPK	Adenosine monophosphate-activated protein kinase
PV	Pemphigus vulgaris
Dsg1	Desmoglein-1
Dsg3	Desmoglein-3
IgG	Immunoglobin G
C.acnes	Cutibacterium acnes
ALA-PDT	5-aminolevulinic acid-photodynamic therapy
LS	Lesional skin
NLS	Non lesional skin
BMI	Body Mass Index
PTEN	Phosphatase and TENsin homolog deleted on chromosome 10
CTCL	Cutaneous T-cell Lymphoma
MF	Mycosis Fungoides
OS	Overall survival
SS	Sezary Syndrome
HIF1a	Hypoxia-inducible factor-1α
GLUT3	Glucose transporter 3
LDHA	Lactate dehydrogenase A
HK2	hexokinase 2
BRAF	v-raf murine sarcoma viral oncogene homolog B1
MEK	Mitogen-activated protein kinase kinase
RTK	Receptor tyrosine kinases
NF-κB	Nuclear factor kappa-light-chain-enhancer of activated B cells
